# Effect of serum anti‐Müllerian hormone levels on the superovulation response in Holstein heifers

**DOI:** 10.1002/vms3.1507

**Published:** 2024-06-19

**Authors:** Mehmet Yildiz, Ilktan Bastan, Sohret Guler, Yunus Cetin

**Affiliations:** ^1^ Department of Obstetrics and Gynecology Faculty of Veterinary Medicine Van Yuzuncu Yil University Van Türkiye; ^2^ Department of Reproduction and Artificial Insemination, Faculty of Veterinary Medicine Burdur Mehmet Akif Ersoy University Burdur Türkiye; ^3^ Genetics and Embryo Technologies Application and Research Center Burdur Mehmet Akif Ersoy University Burdur Türkiye; ^4^ Department of Obstetrics and Gynecology Faculty of Veterinary Medicine Burdur Mehmet Akif Ersoy University Burdur Türkiye

**Keywords:** AMH, embryo, endocrinology, reproduction, superovulation

## Abstract

**Background:**

Anti‐Müllerian hormone (AMH) holds potential as a biomarker for assessing the superovulation (SO) response in cattle. Nonetheless, there exists scant information regarding this aspect in the literature concerning dairy heifers. Given this gap, our objective is to explore the viability of AMH as an indicator for gauging the SO response specifically in Holstein heifers. Furthermore, our aim encompasses examining the variations in AMH levels within the same individuals before and after undergoing SO.

**Methods:**

The study included 41 Holstein heifers. All heifers were superovulated and blood samples were taken both before and after the SO protocol.

**Results:**

The findings revealed that the mean values of serum AMH concentrations before and after SO were 0.122 ng/mL (0.093–0.248 ng/mL) and 0.119 ng/mL (0.084–0.170 ng/mL), respectively. AMH concentrations in heifers were stratified into low (<0.106 ng/mL), medium (0.107–0.126 ng/mL) and high (>0.127 ng/mL) categories both before and after SO.

**Conclusions:**

There was no significant correlation between AMH levels in the heifers both before and after SO treatment with the number of follicles, corpora lutea, total embryos collected or embryos transferred (*p *> 0.05). Furthermore, this study showed that serum AMH concentrations in Holstein heifers did not change after SO treatment. In this study, as AMH levels in Holstein heifers were in a narrow range, a relationship between AMH and SO response could not be determined. In future studies, we believe that it would be more useful to plan more studies in Holstein donor heifers, taking into account the number of animals and AMH levels.

## Graphical abstract

This study showed that serum AMH concentrations in Holstein heifers did not change after SO treatment. Furthermore, in this study, as AMH levels in Holstein heifers were in a narrow range, a relationship between AMH and SO response could not be determined. In future studies, we believe that it would be more useful to plan more studies in Holstein donor heifers, taking into account the number of animals and AMH levels.

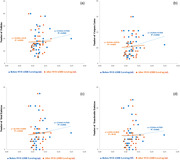

## 1 INTRODUCTION

In the embryonic stage, thousands of oocytes are generated in the ovaries of female calves at birth. However, only a minute proportion undergoes full development and eventually ovulates. The potential for oocyte development can be significantly augmented through superovulation (SO) treatment, a technique frequently employed in tandem with embryo transfer (Mori, 1999). Executing SO in a single cycle facilitates the generation of a larger number of progeny from a donor possessing exceptional genetic attributes (Gadisa et al., 2019; Rocha & Hernando, 2005).

In recent years, there has been a significant upsurge in commercial interest surrounding embryo production, leading to a remarkable increase in the quantity of embryos generated both in vivo and in vitro. The annual production has now reached approximately two million embryos, underscoring the growing significance and demand for assisted reproductive technologies across various industries and sectors. According to a 2022 survey conducted by the International Embryo Technology Association (IETS), the global production totalled 1907,392 embryos. Among these, 386,374 (20.3%) were derived from in vivo methods, whereas 1521,018 (79.7%) originated from in vitro methods. However, despite the notable rise in the production of in vitro‐generated embryos, the utilization of in vivo‐derived embryos remains prevalent (Viana, 2022).

Despite substantial progress in embryo transfer technology, the average count of transferable embryos per SO and collection process has shown little change over the last four decades, maintaining an average of six transferable embryos per SO treatment. This stability can be attributed to significant disparities in the follicle‐stimulating hormone (FSH) responses among donor animals (Monniaux et al., 2010; Rico et al., 2009). When there is a limited number of transferable embryos per procedure or a lack of response to SO treatments, the use of embryo transfer technology becomes cost‐prohibitive. In Holstein cattle, approximately 25% of cows do not exhibit a response to SO treatment (Lerner et al., 1986).

The antral follicle count stands as a crucial parameter in anticipating the response to SO. Typically, ultrasonographic examinations are conducted to gauge antral follicle numbers. However, practical implementation introduces several hurdles, encompassing discrepancies in ultrasound machine specifications, operator proficiency, criteria for tallying antral follicles and the specific phase of the follicular wave during assessment (Monniaux et al., 2010; Singh et al., 2004; Souza et al., 2015). In real‐world settings, accurately identifying cows with the highest potential for embryo production can be formidable due to the cumulative impact of these variables (Ireland et al., 2007).

An alternative method for assessing antral follicles involves measuring serum anti‐Müllerian hormone (AMH) levels (Baldrighi et al., 2014). In cows, the granulosa cells of small antral or preantral follicles exhibit the highest concentrations of AMH. However, as follicular development advances, the levels of AMH decrease (Baarends et al., 1995). AMH is recognized as a biomarker for forecasting the ovarian response to follicular stimulation, particularly in terms of oocyte retrieval, fertilized embryos and transferable embryos (Hirayama et al., 2017; Mossa et al., 2017). Additionally, AMH levels have shown promise as an endocrine marker for predicting superovulatory responses in cows (Rico et al., 2012). However, according to the current literature, no study has been found to indicate the effect of SO treatment on AMH levels.

Indeed, studies examining the potential correlation between AMH levels and SO responses have yielded varying results (Nawaz et al., 2018; Rajesh et al., 2023). Therefore, this study aimed to investigate the relationship between AMH levels and SO response (the number of follicles, corpora lutea [CL] and total embryos collected), as well as the effect of SO treatment on AMH levels in Holstein heifers.

## 2 MATERIALS AND METHODS

### 2.1 Animals

This study was carried out at Burdur Mehmet Akif Ersoy University Faculty of Veterinary Medicine Large Animal Farm (MAKU THG). A total of 41 adult Holstein heifer donors from the this dairy herd were included in the study. The body condition score (BCS) was assessed by a skilled recorder using a scale ranging from 1 to 5, where 1 indicates underweight and 5 denotes obesity, following the methodology outlined by Kasimanickam et al. (2020). All heifers used in the study had BCS between 2.5 and 3.5. Body weights ranged from 380 to 450 kg (401.61 ± 12.23 kg). The heifers participating in the study were used as donors for the first time. Heifers were examined in detail and healthy animals participated in the study. The study was conducted under optimum conditions in accordance with our hypothesis. Animals that displayed three or more CL during transrectal ultrasonography at the time of embryo collection were categorized as responders to the SO treatment (*n* = 36 out of 41 heifers). When the first blood samples were collected, the average age of the heifers was 14 ± 1.05 months before SO treatment. They underwent routine genital examinations, including both rectal and ultrasound examinations. The selected heifers had experienced at least one oestrus cycle and were considered sexually mature. Their ration was formulated according to a concept outlined by the National Research Council (NRC). Furthermore, all procedures conducted in this experiment received approval from the Animal Experiments Local Ethics Committee at Burdur Mehmet Akif Ersoy University Rectorate (Ethical confirmation number: 733).

### 2.2 Collection and storage of blood samples

Blood samples from 41 heifers were collected twice, before and after SO, in sterile vacuum tubes without anticoagulant. Regardless of the stage of the heifer's oestrus cycle, the first batch of blood samples were collected from sexually mature heifers approximately 1 month before SO treatment. The second batch of samples were collected on 7 day of oestrus after SO in donor heifers. After collection, the samples were allowed to clot at 4°C for 20 min, followed by centrifugation at 3000 × *g* for 20 min. The resulting serum was then cautiously collected and stored at −20°C until further analysis. Both blood samples collected before SO and blood samples taken after SO were analysed.

### 2.3 Superovulation protocol

Heifers were subjected to reproductive exam by palpation per rectum before SO treatment. Heifers with palpable CL were selected and administered with an injection of PGF2α (Estrumate (250 μg/mL Cloprostenol) 2 mL intramuscular, Merck Animal Health) to cause luteolysis and to induce oestrus. Detection of oestrus was made by routine rectal palpation, ultrasound examination and clinical observation. SO protocol was initiated between 8 and 10 days after oestrus (Center et al., 2018). The SO was achieved with GnRH (Gestavet GnRH (Gonadorelin 0.1 mg/mL) 1 mL intramuscular), intravaginal progesterone (P4) implants (Prid‐Delta; Ceva Sante Animale), FSH (Stimufol, Reprobiol, Belgium) intramuscular, in decreasing doses, and PGF2α (Dinolytic HC, Zoetis).

The donor animals were first administered intravaginal progesterone and concurrent intramuscular GnRH treatments, and the day of administration was recorded as day 0. Following a 48‐h period, the heifers were administered with decreasing doses of FSH twice daily, with a 12‐h interval, over a 4‐day period. Heifers were injected (i.m.) with 1.5 mL of FSH twice daily, then 1, 0.5 and 0.5 mL on days 3–5, respectively. Two doses of PGF2α were administered on d5, 12 h apart, and the CIDR was removed concurrently with the first dose of PGF2α administration. GnRH of 100 μg was administered in the evening of d6. On day 7, the heifers were examined by transrectal ultrasonography (Hasvet 838, Hasvet Medical) to determine the number of follicles on the ovaries and inseminated twice, 12 h apart. Embryos were recovered on day 14.

### 2.4 Embryo collection and evaluation

On the 7th day, ultrasound examination was employed to determine the count of ovulatory‐sized follicles, defined as those with a diameter of ≥10 mm. Following this assessment, artificial insemination was carried out. Subsequently, on 7 day after artificial insemination, ultrasound imaging was utilized to count the number of CLs.

After the ovarian ultrasound evaluation, epidural anaesthesia (administered using Adokain from Sanovel) was administered. The retrieval of embryos was conducted using a 2‐way flushing equipment. The uterus was flushed with approximately 1.5 L of lactated Ringer solution, with 750 mL allocated for each uterine horn. This flushing solution consisted of 1.5% foetal bovine serum and 0.1% antibiotic.

The retrieved embryos were collected onto an embryo filter and promptly transferred to a petri dish. Under a stereo microscope, a thorough examination was conducted, and the embryos were assessed for quality based on the criteria set forth by the International Embryo Transfer Society (Stringfellow & Givens, 2010). The quality assessment was graded on a scale ranging from 1 (excellent or good), 2 (fair), 3 (poor) to 4 (dead or degenerating).

Following the rinsing process, 2 mL of PGF2α was administered intramuscularly, along with intrauterine antibiotic treatment to forestall pregnancy and uterine infections. These measures were implemented to ensure the well‐being of the animals and to minimize any potential complications.

### 2.5 Anti‐Müllerian hormone analysis

Various measurement methods can be employed to determine AMH levels (Koca et al., 2023; Widodo et al., 2022). The commercially available human AMH Gen II ELISA kit (Beckman‐Coulter Inc.) was used to measure serum AMH concentrations in cattle per kit instructions (Hirayama et al., 2019; Scarlet et al., 2021). This AMH kit was validated to be used for bovine by Ireland et al. (2008) for the first time. AMH analysis was conducted on all blood samples obtained both before and after SO treatment. In other words, a total of 82 blood samples, collected from 41 heifers, were individually analysed. This specific ELISA was designed with a limit of detection set at 0.05 ng/mL. The coefficients of variation associated with the assay were ≤10.3%, indicating good precision and reproducibility in measuring AMH concentrations.

### 2.6 Statistical analysis

Statistical analyses for the data obtained in this study were carried out using the licensed SPSS 25 software. Whether the data conformed to normal distribution was evaluated with the Kolmogorov–Smirnov normality test. When analysing differences among the groups, the ANOVA test was utilized, given that the variables adhered to a normal distribution. To compare AMH concentrations in the serum samples collected before and after SO, a dependent sample t test was conducted. The relationships between serum AMH concentrations of each heifer before and after SO and the number of follicles, CLs total embryos retrieved, and embryos transferred were evaluated using the Pearson correlation coefficient. All results are presented as means ± standard error. A *p*‐value of <0.05 was regarded as statistically significant.

## 3 RESULTS

The findings revealed that the mean values of serum AMH concentrations before and after SO were 0.122 ng/mL (0.093–0.248 ng/mL) and 0.119 ng/mL (0.084–0.170 ng/mL), respectively. The SO treatment did not lead to a significant difference in the AMH levels of the heifers (*p* > 0.05) (Table 1).

**TABLE 1:** Comparison of anti‐Müllerian hormone (AMH) levels measured before and after superovulation (SO) treatment.

	*N*	Mean ± SEM	Min. statistic	Max. statistic	*t*	*p*
Before SO AMH, ng/mL	36	0.122 ± 0.005	0.093	0.248	0.565	0.576
After SO AMH, ng/mL	36	0.119 ± 0.003	0.084	0.170		

Abbreviation: SEM, standard error mean.

**FIGURE 1:**
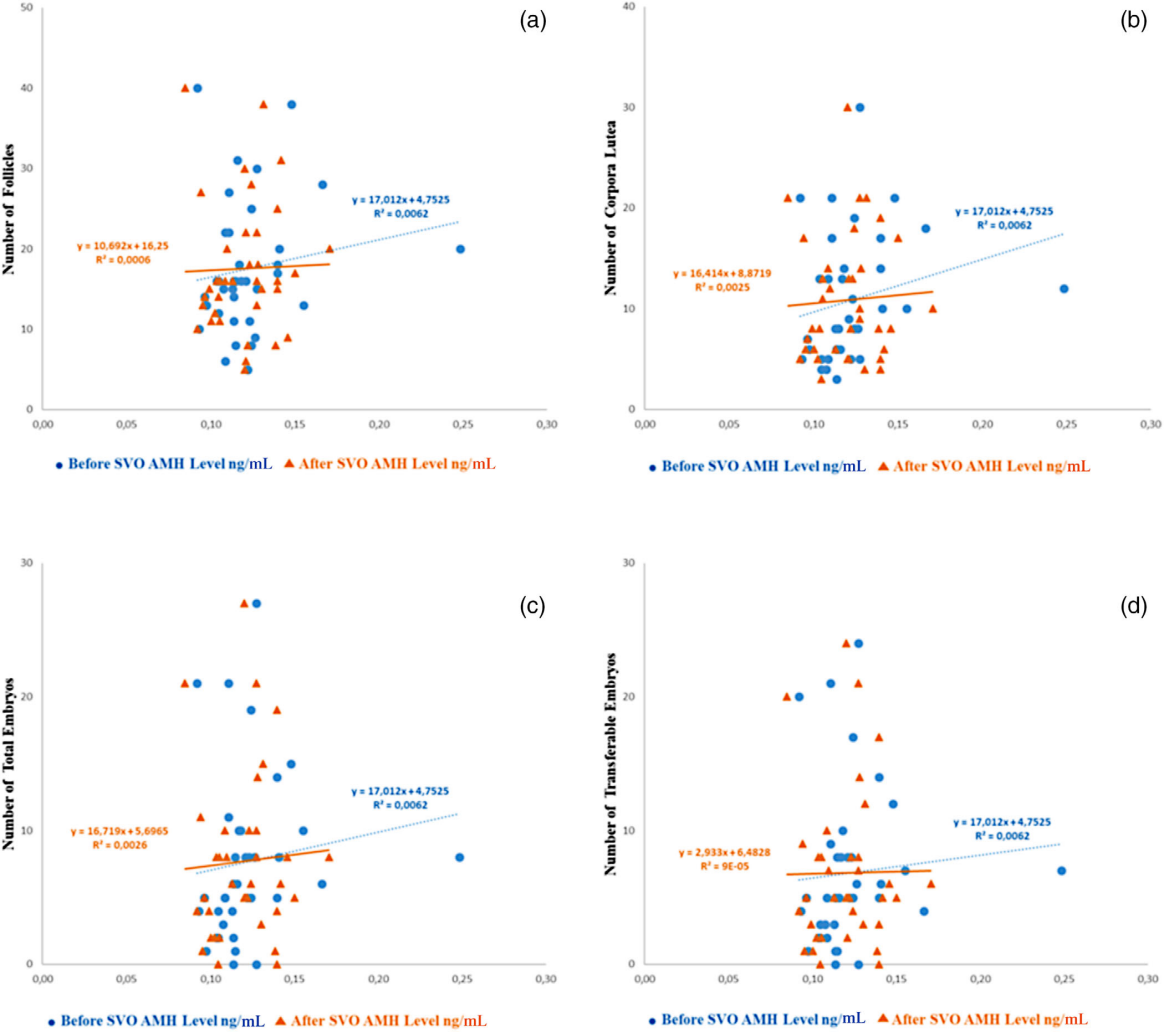
Relationship between anti‐Müllerian hormone (AMH) levels and before and after superovulation (SO) treatment. Numbers of follicles (a), corpora lutea (b), total embryos (c) and transferable embryos (d) in relation to AMH levels before and after SO treatment are depicted.

For each heifer, no significant correlations were identified between before and after SO treatment serum AMH concentration and the number of follicles (*r* = 0.147, *r* = 0.25, *p* > 0.05), CL (*r* = 0.222, *r* = 0.50, *p* > 0.05), total recovered embryos (*r* = 0.130, *r* = 0.092 *p* > 0.05) and transferable embryos (*r* = 0.209, *r* = 0.281, *p* > 0.05) (Figure 1). Moreover, Figure 1 shows findings of a regression analysis.

The samples were categorized based on Hirayama et al. (2017) into three groups before SO: those with the lowest 25% concentration, medium concentration and the highest 25% concentration. Before SO treatment, AMH concentration levels were classified as follows: low (*n*: 9; <0.106 ng/mL), medium (*n*: 18; 0.107–0.126 ng/mL) and high (*n*: 9; >0.127 ng/mL) groups (Table 2). The results obtained when the AMH levels of the after SO heifers were grouped into low, medium and high according to the pre‐SO thresholds are presented in Table 3. No significant difference was observed in superovulatory response and embryo collection results when AMH levels of heifers before and after SO treatment were grouped into low, medium and high categories.

**TABLE 2:** Before superovulation (SO) anti‐Müllerian hormone (AMH) concentrations and the relationship between SO response.

	Before SO AMH levels (ng/mL)			
	Low (0.093–0.106)	Medium (0.107–0.126)	High (0.127–0.248)	
Donors (no.)	9	18	9	*p*
Before SO AMH, ng/mL	0.10 ± 0.01^a^	0.12 ± 0.01^a^	0.15 ± 0.1^b^	<0.001
Follicles (no.)	17.56 ± 3.02	16.39 ± 1.87	19.78 ± 2.88	0.616
Corpora lutea (no.)	8.67 ± 1.94	10.95 ± 1.64	12.78 ± 1.72	0.392
Total embryos recovered (no.)	5.22 ± 2.03	8.67 ± 1.68	9.25 ± 1.26	0.329
Transferable embryos (no.)	5.00 ± 1.93	7.78 ± 1.59	8.25 ± 1.46	0.455
Degenerated embryos (no.)	0.11 ± 0.11	0.89 ± 0.25	1.00 ± 0.33	0.084

*Note*: Means ± SEM within rows with different superscripts (a, b) are significantly different (*p *< 0.05).

**TABLE 3:** After superovulation (SO) anti‐Müllerian hormone (AMH) concentrations and the relationship between SO response.

	After SO AMH levels (ng/mL)			
	Low (0.084–0.106)	Medium (0.107–0.126)	High (0.127–0.248)	
Donors (no.)	12	10	14	*p*
After SOV AMH, ng/mL	0.10 ± 0.01^a^	0.12 ± 0.01^b^	0.14 ± 0.01^c^	<0.001
Follicles (no.)	16.58 ± 2.48	16.90 ± 2.73	18.79 ± 2.18	0.774
Corpora lutea (no.)	9.17 ± 1.56	12.40 ± 2.40	11.14 ± 1.64	0.486
Total embryos recovered (no.)	5.67 ± 1.68	8.70 ± 2.13	9.38 ± 1.71	0.307
Transferable embryos (no.)	5.25 ± 1.60	7.50 ± 1.96	8.69 ± 1.70	0.359
Degenerated embryos (no.)	0.33 ± 0.19	1.20 ± 0.39	0.69 ± 2.24	0.104

*Note*: Means ± SEM within rows with different superscripts (a, b, c) are significantly different (*p *< 0.05).

## 4 DISCUSSION

Accurately identifying donors is crucial in terms of time and cost efficiency, as well as achieving a successful SO response. Recent studies in veterinary medicine have demonstrated that AMH concentration stands as a dependable phenotypic marker for assessing ovarian reserve size, ovarian function, SO response, as well as fertility and herd longevity (Nawaz et al., 2018). Nonetheless, certain researchers have reported no discernible correlation between AMH and SO in cows (Rajesh et al., 2023). In this study, there was no significant correlation between AMH levels in the heifers both before and after SO treatment with the number of follicles, CL, total embryos collected or embryos transferred. Furthermore, this study showed that serum AMH concentrations in Holstein heifers did not change after SO treatment.

Consistency in AMH levels has been noted in dairy cows, observed within the same oestrous cycle (Rico et al., 2009; Souza et al., 2015), across various days in separate cycles (Rico et al., 2009) and in both naturally occurring and synchronized oestrous cycles (Pfeiffer et al., 2014). In this study, we observed similar results as there were no significant differences in AMH concentrations between blood serum samples collected at different stages of the oestrous cycle before and after SO treatment. Therefore, our study demonstrates the reproducibility of AMH levels within the same animals across different time points.

AMH levels exhibit significant variation among dairy cattle (Koca et al., 2024). Center et al. (2018) documented a wide range of plasma AMH concentrations in cows, spanning from 0.013 to 0.898 ng/mL, with an average AMH level of 0.293 ng/mL. Saltik & Cetin (2020) conducted a study encompassing both heifers and cows, revealing a spectrum of AMH concentrations ranging from 0.001 to 0.700 ng/mL. Furthermore, investigations on heifers have shown that AMH levels fluctuate between approximately 0.05 and 0.50 ng/mL (Ireland et al., 2008). In our research, we found that AMH concentrations in serum samples from 41 different heifers fell within a range similar to that reported in these studies, varying from 0.084 to 0.248 ng/mL. The average AMH level across all heifers was measured to be 0.122 ng/mL. Moreover, in our study, AMH levels were found to be in a narrow range compared to the levels reported in previous studies.

The reliability of AMH as an endocrine marker for predicting SO responses in cows was investigated by Rico et al. (2012). Previous studies have demonstrated positive correlations between plasma AMH levels and various reproductive parameters in cows. Rico et al. (2012) reported positive associations between plasma AMH levels and the presence of large follicles at oestrus (*r* = 0.46), as well as the number of CL (*r* = 0.43) in superovulated cows. Similarly, Center et al. (2018) observed positive relationships between circulating AMH levels and several reproductive parameters. They noted a positive correlation between AMH levels and the number of follicles at the start of SO (*r* = 0.458). Furthermore, they found positive associations between AMH levels and the count of CL (*r* = 0.452), as well as the number of embryos collected during uterine flushing (*r* = 0.430). Center et al. (2018) reported a wide range of AMH concentrations, from 0.013 to 0.898 ng/mL. In the study conducted by Rico et al. (2012), Holstein cows had a wide range of AMH concentrations from 0.005 to 0.244 ng/mL. Notably, values below 0.087 ng/mL were associated with an inadequate SO response, characterized by less than 15 large follicles during oestrus. Souza et al. (2015) proposed a cut‐off value of 0.130 ng/mL AMH for identifying poor responders to SO based on similar criteria. In contrast to these findings, our study did not reveal significant correlations between individual circulating AMH concentrations and parameters such as follicle count, CL count, total embryos recovered or transferable embryos. Interestingly, the serum AMH concentrations in this study displayed a narrower range compared to those observed in previous investigations. Most of the donor heifers utilized in our study were closely related to maternal or paternal lineages of second or third generations. If the AMH levels are inherited from the mother and father, the blood relationships between the heifers may have caused the AMH levels to be distributed within a narrow range compared to other studies. Nawaz et al. (2018) determined the genomic heritability of AMH. Furthermore, the relationship between AMH and reproductive variables are not always linear. In relation to this finding, it was observed that the concentration of AMH exhibited a quadratic correlation with the reproductive performance of dairy cows. Furthermore, cows with both minimal and maximal levels of AMH displayed inferior fertility compared to their counterparts with moderate levels of AMH (Akbarinejad et al., 2020). Given this information, as highlighted in our study, it appears that establishing a relationship with the SO response may not be established in donors exhibiting a narrow AMH reference range.

Addressing this subject, Center et al. (2018) categorized circulating AMH concentrations into quartiles (Q1–Q4). Their findings indicated that donor cows in the highest quartile (Q4) of AMH levels exhibited a greater number of follicles and CL during SO treatments compared to cows in Q1 or Q2 (*p* < 0.001). However, no statistically significant differences were observed between AMH concentrations and follicle counts, CL counts or the number of total embryos in the Q1 (0.013–0.168 ng/mL) and Q2 (0.169–0.263 ng/mL) groups. Similarly, Souza et al. (2015) divided circulating AMH concentrations into quartiles (Q1–Q4). Their results suggested that donor cows in the highest quartile (Q4) of AMH levels had a greater number of CL during SO treatments compared to the cows in Q1 and Q2 (*p* < 0.01). Nevertheless, no statistically significant differences were noted between the AMH concentration and CL counts or the number of embryos produced in the Q1 (0.001–0.082 ng/mL) and Q2 (0.091–0.132 ng/mL) groups. Notably, AMH levels exhibit a wide range across different studies. We found that AMH levels had a narrower range (0.084–0.248 ng/mL), possibly due to familial relationships among the heifers in our study. Despite categorizing the AMH levels into three groups, no significant differences were found between these groups concerning AMH levels, follicle counts, CL numbers or the total number of embryos (*p* > 0.05).

## 5 CONCLUSION

There are two main findings from the study. First, the SO treatment did not alter the AMH levels in the Holstein heifers, which remained at a constant level. Second, our research has shown that there is no discernible relationship between AMH and the SO response when the AMH levels of the participating Holstein heifer donors are within a narrow reference range. Based on the information given, the narrow range of AMH levels in Holstein heifers in this study suggests that there may not be a relationship between AMH and SO response. Furthermore, there exists a necessity for determining AMH reference ranges for Holstein heifers. In future studies, we believe that it would be more useful to plan more studies in Holstein donor heifers, taking into account the number of animals and AMH levels.

## AUTHOR CONTRIBUTIONS

**Mehmet Yildiz**: Conceptualization; data curation; investigation; methodology; software; validation; visualization; writing‐original draft; writing‐review and editing. **Ilktan Bastan**: Data curation; investigation; methodology; software; writing‐original draft; writing‐review and editing. **Sohret Guler**: Data curation; investigation; writing‐original draft; writing‐review and editing. **Yunus Cetin**: Conceptualization; funding acquisition; investigation; methodology; project administration; resources; writing‐original draft; writing‐review and editing; supervision. All authors also reviewed and approved final version of manuscript.

## CONFLICT OF INTEREST STATEMENT

None of the authors have any conflicts of interest to declare.

## ETHICS STATEMENT

All procedures conducted in this experiment received approval from the Animal Experiments Local Ethics Committee at Burdur Mehmet Akif Ersoy University Rectorate (Ethical confirmation number: 733).

## Data Availability

The datasets used and analysed during the current study are available from the corresponding author on request.
